# Photoelectrochemical Performance of Nanotubular Fe_2_O_3_–TiO_2_ Electrodes under Solar Radiation

**DOI:** 10.3390/nano12091546

**Published:** 2022-05-03

**Authors:** Monika Sołtys-Mróz, Karolina Syrek, Łukasz Pięta, Kamilla Malek, Grzegorz D. Sulka

**Affiliations:** Faculty of Chemistry, Jagiellonian University, Gronostajowa 2, 30-387 Krakow, Poland; lukasz.pieta@doctoral.uj.edu.pl (Ł.P.); kamilla.malek@uj.edu.pl (K.M.); sulka@chemia.uj.edu.pl (G.D.S.)

**Keywords:** anodic titanium oxide, hematite, electrochemical deposition, photoelectrochemical activity, glucose sensor

## Abstract

Fe_2_O_3_–TiO_2_ materials were obtained by the cathodic electrochemical deposition of Fe on anodic TiO_2_ at different deposition times (5–180 s), followed by annealing at 450 °C. The effect of the hematite content on the photoelectrochemical (PEC) activity of the received materials was studied. The synthesized electrodes were characterized by field emission scanning electron microscopy (FE-SEM), energy dispersive spectroscopy (EDS), X-ray diffraction (XRD), Raman spectroscopy, diffuse reflectance spectroscopy (DRS), Mott–Schottky analysis, and PEC measurements. It was shown that the amount of deposited iron (ca. 0.5 at.%–30 at.%) and, consequently, hematite after a final annealing increased with the extension of deposition time and directly affected the semiconducting properties of the hybrid material. It was observed that the flat band potential shifted towards more positive values, facilitating photoelectrochemical water oxidation. In addition, the optical band gap decreased from 3.18 eV to 2.77 eV, which resulted in enhanced PEC visible-light response. Moreover, the Fe_2_O_3_–TiO_2_ electrodes were sensitive to the addition of glucose, which indicates that such materials may be considered as potential PEC sensors for the detection of glucose.

## 1. Introduction

In recent years, there is a growing interest in the connecting of two semiconductors (generally, TiO_2_ and Fe_2_O_3_) both exhibiting properties such as good chemical stability, non-toxicity, light absorption, and appropriate band alignment [1,2,3,4,5] as this allows their use in the preparation of hybrid materials that absorb radiation of a wide range of wavelengths. Among the methods of combining these oxides, electrodeposition has attracted attention because of its simplicity, affordability, and in most cases, the reasonable speed and easy control of its operating conditions [6,7]. This synthesis method can be carried out as anodic [8], cathodic [8], and pulsed/potential cycling electrodeposition [9]. It is widely recognized that electrodeposition conditions such as the composition of the deposition bath, its pH, temperature, applied potential, and the process duration affect the formation of different surface morphologies, which in turn might impact the photoelectrochemical (PEC) activity of nanostructured electrodes [10].

The summary of nanostructured hybrid materials based on Fe/Fe_2_O_3_ and TiO_2_ synthesized by electrochemical deposition is presented in Table 1.

For instance, Mohapatra et al. [11] obtained Fe_2_O_3_–TiO_2_ composites by using the pulsed technique for electrodeposition of iron inside TiO_2_ nanotubes, followed by high-temperature transformation of deposited Fe into Fe_2_O_3_ in an oxygen atmosphere. It was found that by changing the applied current density, deposition time, and duty cycle, Fe_2_O_3_ nanorods with a diameter of 80–90 nm and a length of up to 550 nm could be obtained within anodic TiO_2_ nanotubes. Moreover, Liang et al. [12] deposited two types of hematite nanoparticles on the TiO_2_ surface: sphere-like large particles (500 ± 10 nm) and sub-particles with a smaller diameter. The distribution density of sub-particles at the TiO_2_ surface increased when the higher potential was applied, and their shape and size were controllable by the applied potential and time. On the other hand, Jeon et al. [13] proposed a combination of two techniques, (i) impregnation of anodic TiO_2_ in an iron (III) chloride solution and (ii) electrochemical deposition, to obtain Fe_2_O_3_–TiO_2_. It was found that 1 h immersion followed by potential cycling in an electrolyte containing 5 mM sodium fluoride, 0.1 M sodium chloride, 1 M hydrogen peroxide, and 5 mM iron (III) chloride causes the formation of hematite particles of 20–30 nm in diameter at the mouth of TiO_2_ nanotubes, while 24 h immersion resulted in completely filling of the interior of the nanotubes, and a cap top layer was even formed. What is more, the obtained hybrid materials were tested for PEC water splitting. The improved PEC properties (1 mA cm^−3^ at 1.0 V vs. SCE in 0.1 M Na_2_SO_4_, under visible light) were observed for the material immersed for 24 h before electrodeposition. Cong et al. [14] used a similar strategy to deposit Fe_2_O_3_ nanoparticles (with an average diameter of 35 nm) at the mouths, tube walls, and bottoms of TiO_2_ nanotubes. The synthesis procedure included a few alternating cycles of soaking TiO_2_ nanotubes in a 0.05 M ferric nitrate aqueous solution for 10 min, and electrochemical reduction in a supporting electrolyte containing sodium sulfate. Furthermore, Cong et al. [15] compared the above-obtained materials with anodic TiO_2_ samples impregnated in an aqueous suspension of 5 mg cm^−3^ α-Fe_2_O_3_ nanoparticles. It was observed that the material obtained by electrodeposition showed a higher distribution density of smaller hematite particles on the surface, which may be responsible for the enhanced PEC properties. The electrodeposited samples showed a higher phenol removal efficiency and better visible light response. What is more, it was shown that such a modification improved the interfacial charge transport of the TiO_2_ nanotubes. Ren et al. [16] used an electrodeposition method to obtain Fe–TiO_2_ hybrid materials for PEC and photocatalytic applications. As reported, with increasing bias voltages from 0.5 to 2.0 V vs. Ag/AgCl (3 M KCl), the average photocurrent densities under UV and visible light irradiation measured in a 1 M urea solution increased from 17.65 to 201.85 mA m^−2^, respectively. On the other hand, Tsui et al. [17] noticed that for a prolonged deposition time, more Fe_2_O_3_ nanoparticles cover the surface of the TiO_2_ nanotubes, and, as a consequence, the UV and visible light PEC efficiencies increased to 5% and 1%, respectively. Additionally, Chin et al. [18] studied the effect of the applied potential during the electrodeposition of iron on the visible light absorption of Fe–TiO_2_ hybrid materials. It was shown that for higher applied potentials (3 or 4 V), lower light adsorption was observed due to the full coverage of the mouth of the TiO_2_ nanotubes by the Fe particles, which drastically influenced the PEC properties.

It is worth highlighting that the comprehensive characterization of semiconducting properties (such as band gap energy, flat band potential, charge density, and conduction band potential) is an important aspect of the study of photoanode materials. What is more, it should be emphasized that those properties (e.g., donor density and flat band potential [19]) of the anodic TiO_2_ substrate itself are affected by different oxide thicknesses, pore sizes, wall thicknesses [20], and post-heat-treatment conditions [21,22] due to changes in the oxide morphology, crystallinity, and composition [19,20,21,22,23,24,25,26]. For this reason, a particular emphasis should be put on the systematic study of the semiconducting properties of Fe_2_O_3_–TiO_2_ materials.

Therefore, in this paper, Fe_2_O_3_–TiO_2_ hybrid materials were synthesized by the cathodic electrodeposition of iron at different durations (5–180 s), followed by annealing at 450 °C in air. A complex characterization (SEM/EDS, XRD, Raman spectroscopy, UV-Vis DRS) of the obtained hybrid materials was performed. Additionally, for the first time, a detailed investigation of the semiconducting properties of Fe_2_O_3_–TiO_2_ electrodes was provided. Moreover, the PEC water-splitting experiments were carried out under solar and monochromatic illumination. The obtained results were compared with a similar material received by impregnation of anodic titanium oxide in a 100 mM FeCl_3_ solution, the full characterization of which was previously reported [27,28]. Furthermore, the synthesized materials were also tested for the first time as potential non-enzymatic PEC sensors for the determination of glucose.

## 2. Materials and Methods

### 2.1. Synthesis of Nanostructured Fe_2_O_3_–TiO_2_

**Figure 1:**
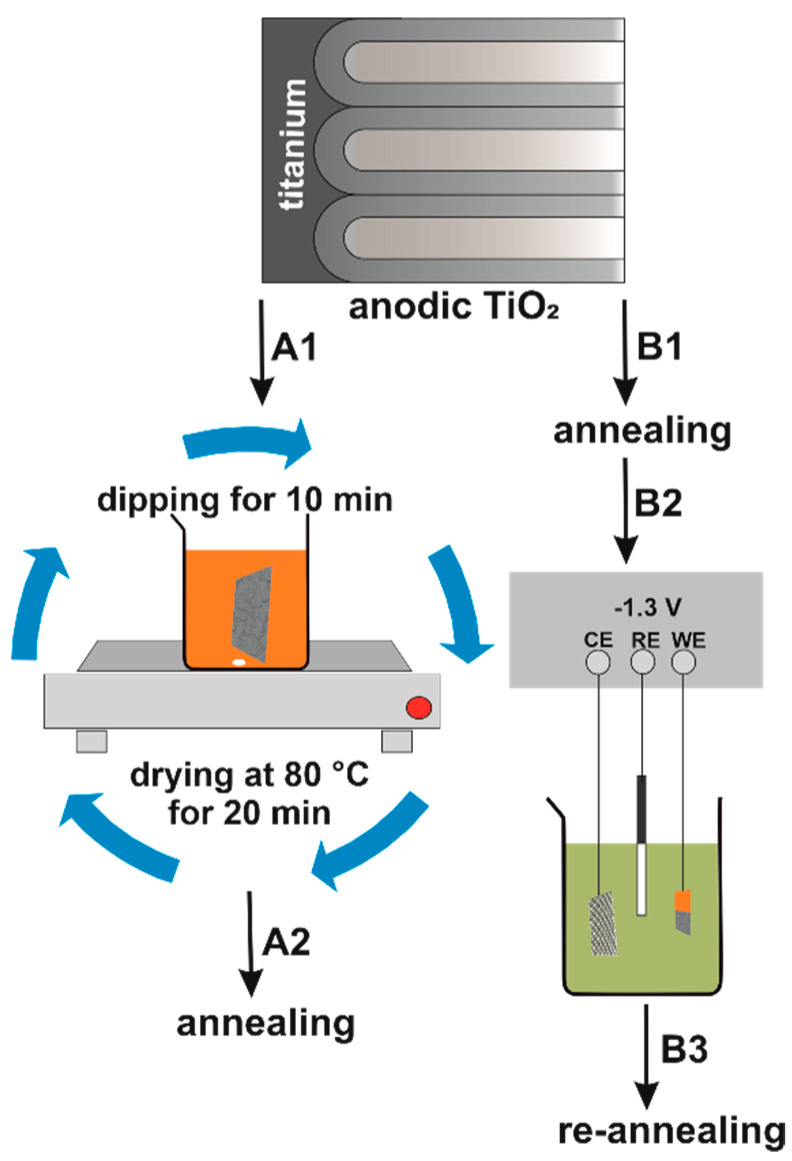
Synthesis of nanostructured Fe_2_O_3_–TiO_2_ by impregnation (**A1**) and electrochemical deposition (**B2**).

Anodic TiO_2_ samples were prepared according to the previously described method [27]. The obtained pore diameter, interpore distance, length, and wall thickness were around 54, 90, 600, and 20 nm, respectively [23,24,25,26]. Figure 1 shows the schematic procedure used for the preparation of the studied and reference samples. The anodic TiO_2_ layers before electrodeposition were annealed at 400 °C for 2 h using a muffle furnace (FCF 5SHM Z, Czylok, Krakow, Poland) with a heating rate of 2 °C min^−1^ (Figure 1B1). Using as-received anodic TiO_2_ layers, Fe was potentiostatically electrodeposited from a mixture of 0.1 M FeSO_4_ (Sigma-Aldrich, India) and 0.1 M H_3_BO_3_ (Chempur, Poland) (pH = 3.9) at −1.3 V vs. SCE for 5–180 s (Figure 1B2) [17]. After deposition, the samples were annealed at 450 °C for 1 h (Figure 1B3). As the reference sample, an anodic TiO_2_ layer impregnated with 100 mM FeCl_3_ (Honeywell, Seelze, Germany) (Figure 1A1) and annealed at 450 °C for 2 h (Figure 1A2) was used. The experimental details have been described previously [27,28].

### 2.2. Characterization of Fe_2_O_3_–TiO_2_ Materials

The morphology and chemical composition of synthesized materials were characterized by using a field emission scanning electron microscope (FE-SEM/EDS, Hitachi S-4700 with a Noran System 7, Tokyo, Japan). The phase composition was determined by using a Rigaku Mini Flex II diffractometer (Rigaku, Tokyo, Japan) with Cu Kα radiation (1.54060 Å) at the 2θ range of 20–60°. Optical band gaps were determined based on UV-Vis Diffuse Reflectance Spectroscopy (DRS) measurements performed at the wavelength range of 200–800 nm (with a step size of 2 nm) using a Lambda 750S spectrophotometer (PerkinElmer, Waltham, MA, USA). The Spectralon^®^ SRS-99-010 diffuse reflectance standard was used as a reference. Raman spectra were collected by using a Raman microscope, WITec Alpha 300 (WITec, Ulm, Germany), equipped with an air-cooled solid-state laser operating at 532 nm, a 600 grooves/nm grating, and a CCD detector. A microscope was coupled with a laser and a spectrograph via a single-mode optical fiber with a diameter of 50 µm. Samples were illuminated with the output laser power of 1.8 mW through a 40× air objective (NA: 0.6). Fifty accumulations with an integration time of 1 s were acquired in the range of 0–4000 cm^–1^, with a spectral resolution of ca. 3 cm^–1^. From 5 to 10 Raman spectra were acquired from randomly selected points on the sample surface, and if the spectra represented a similar spectral profile, they then were averaged.

### 2.3. Electrochemical and Photoelectrochemical Measurements

Electrochemical and photoelectrochemical measurements were performed in a three-electrode system, where a SCE electrode (3 M KCl), platinum wire, and Fe_2_O_3_–TiO_2_ sample were used as a reference, counter, and working electrodes, respectively. Mott–Schottky analyses were carried out using a Gamry Reference 3000 potentiostat (Gamry Instruments, Warminster, PA, USA) in the dark, at the constant frequencies of 200, 500, and 1000 Hz in a 0.1 M KNO_3_ solution (pH = 6.1). Photoelectrochemical tests were carried out using a photoelectric spectrometer (Instytut Fotonowy, Krakow, Poland) equipped with a 150 W xenon arc lamp in a Teflon cell with a quartz window. The photocurrent vs. time curves was recorded in a 0.1 M KNO_3_ (Sigma-Aldrich, Spain) aqueous solution (pH = 6.1) under the applied potentials of 0–1 V vs. SCE (with a 200 mV step). Pulse illumination in the range of 200–600 nm with a 10 nm wavelength step and 15 s light and 10 s dark cycles was used. In addition, PEC properties were investigated using a solar simulator equipped with a 150 W xenon lamp, and an Air-Mass 1.5 G filter (Instytut Fotonowy, Krakow, Poland), which corrects the spectral output of the lamp to match the solar spectrum in the wavelength range of 350–700 nm. The received materials were also tested as glucose sensors, and their chronoamperometric response in the dark and under simulated solar illumination was studied. The tests were carried out at the applied potential of 1 V vs. SCE in a 0.1 M KNO_3_ solution containing 1.06–10.28 mM L^−1^ glucose.

## 3. Results and Discussion

### 3.1. Morphology and Crystallinity of Fe_2_O_3_–TiO_2_ Nanostructured Materials Synthesized by Electrodeposition

**Figure 2:**
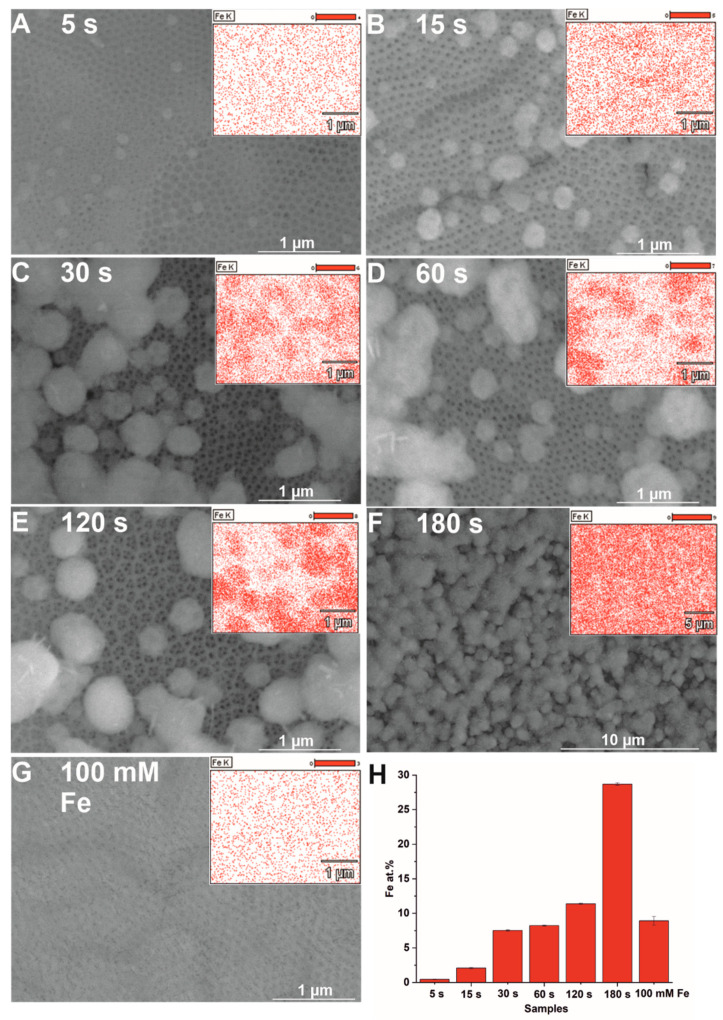
SEM images with EDS maps of Fe distribution (insets) of hybrid Fe_2_O_3_–TiO_2_ samples electrodeposited for 5 s (**A**), 15 s (**B**), 30 s (**C**), 60 s (**D**), 120 s (**E**), and 180 s (**F**), and impregnated in a 100 mM FeCl_3_ solution (**G**). The estimated average iron content for the corresponding modified samples (**H**).

The electrodeposition of iron at the constant potential mode is characterized by a typical exponential decay of current density to a steady-state value (Figure S1A, Supplementary Materials) [12]. What is more, based on current–time transients, the total charge density was obtained (Figure S1B, Supplementary Materials). As expected, the total charge density passing through the system increases with the prolonged deposition, indicating a higher amount of iron deposited on the anodic TiO_2_ surface, which is consistent with the data in the literature [29]. To confirm it, SEM imaging of the prepared samples (Figure 2A–F) with EDS mapping was performed. It can be observed that hematite particles resulting from Fe deposition and annealing have a diameter of ca. 150 nm and are unevenly distributed over the porous surface. The estimated Fe content (Figure 2H) gradually increases from about 0.50 at.% to nearly 30 at.% as deposition time increases from 5 s to 180 s. Moreover, the iron content determined for the impregnated sample (Figure 2G) was comparable to that of the sample electrodeposited for 60 s (Figure 2D), for which the Fe content was about 7.5 at.%. It can be concluded that electrodeposition using anodic TiO_2_ layers as substrates results in the formation of a more controllable distribution of particles over the oxide layer when compared to the sample formed by impregnation.

**Figure 3:**
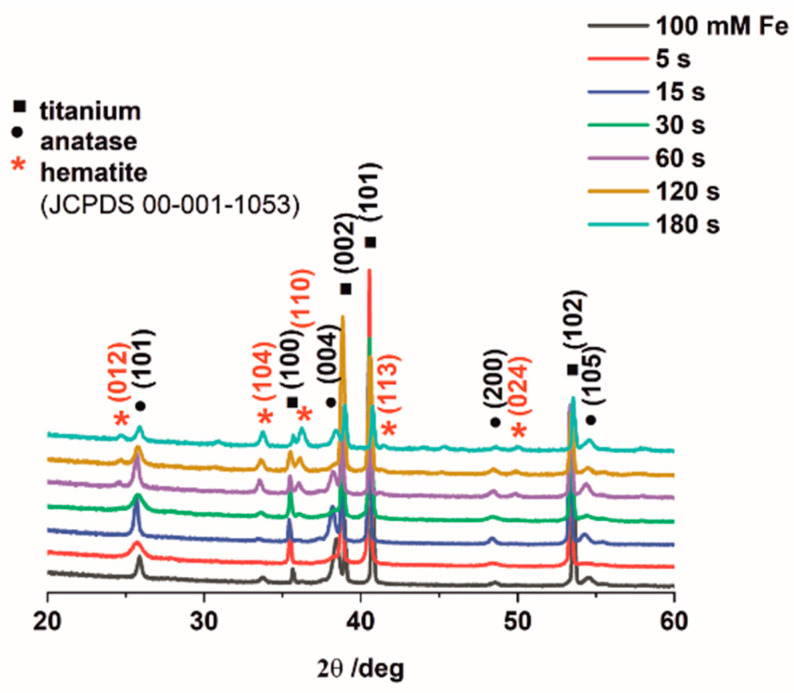
XRD patterns of all studied Fe_2_O_3_–TiO_2_ materials.

X-ray diffraction measurements were performed to characterize the crystal structure of obtained samples. The XRD patterns (Figure 3) of the prepared materials exhibited three main components. The main diffraction peaks correspond to the reflection from (100), (002), (101), (102) titanium (JCPDS card no. 05-0682) crystal planes and (101), (004), (200), (105) anatase (JCPDS card no. 21-1272) crystal planes. It can be seen that hematite peaks were not observed for the sample electrodeposited for 5 s due to a very low quantity of iron (ca. 0.5 at.%). However, for the samples electrodeposited for longer durations, such as 15 s and 30 s, (104), (110) crystal planes of rhombohedral hematite (JCPSD card no. 00-001-1053) are clearly visible. When compared with the impregnated sample, the materials electrodeposited for 60 s, 120 s, and 180 s, all having much higher Fe contents, revealed the presence of additional crystal planes of (012), (113), (024) that correspond to hematite.

**Figure 4:**
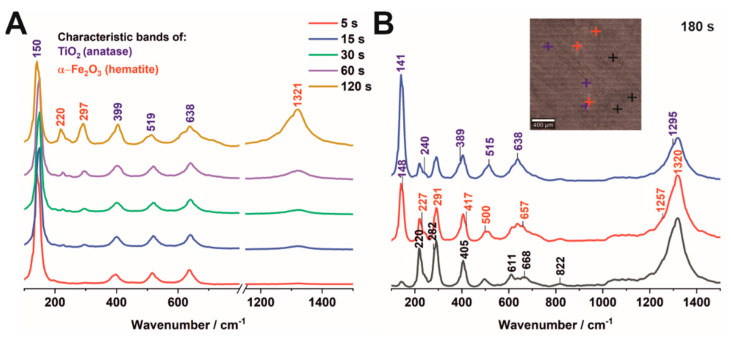
Raman spectra of the Fe_2_O_3_–TiO_2_ samples obtained by electrodeposition for 5–120 s (**A**) and 180 s (**B**). The colors of different areas on the picture correspond to the colors of the Raman spectra.

Figure 4 displays the Raman spectra acquired for hybrid Fe_2_O_3_–TiO_2_ samples obtained by electrodeposition for 5–120 s (A) and 180 s (B). Regardless of the amount of hematite obtained after annealing, all samples are composed of anatase and hematite, as manifested by Raman bands for TiO_2_ at 150, 399, 519, and 638 cm^−1^ [25,28] as well as for α-Fe_2_O_3_ at 220, 284, and 1318 cm^−1^ [27]. In turn, an intensity ratio of the α-Fe_2_O_3_ and TiO_2_ bands clearly shows that the content of hematite gradually increases, and only these iron species are formed when anodic TiO_2_ is subjected to electrodeposition of up to 60 s. The intensity of the α-Fe_2_O_3_ features varies slightly across the sample surface, indicating that its growth is uneven (Figure S2, Supplementary Materials). After 120 s, additional low- and medium-intensity Raman bands appear at ca. 230 (goethite, FeOOH), 390/500 (maghemite, γ-Fe_2_O_3_), 610 (wüstite, FeO), 660 (magnetite, Fe_3_O_4_), and 1300 (lepidocrocite, γ-FeO(OH)) cm^−1^ (Figure 4A) [28]. The distribution of these iron oxides changes due to the elongation of the deposition time up to 180 s (Figure 4B). Here, there are sites in which the hematite/anatase ratio alters from low (blue trace) to high (red trace), while the contribution of other iron oxides is similar.

### 3.2. Semiconducting Properties

**Figure 5:**
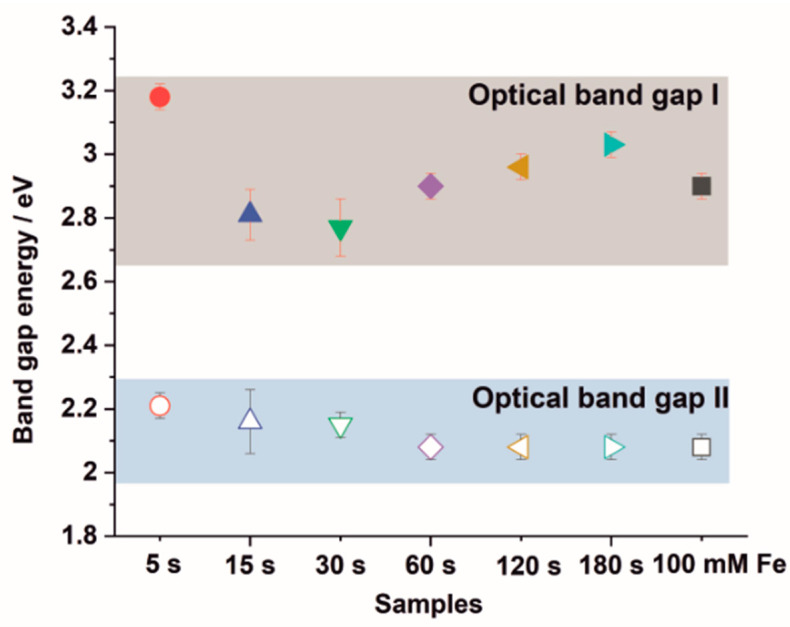
Band gap energies estimated from UV–Vis DRS measurements for all tested Fe_2_O_3_–TiO_2_ materials.

For all studied samples, optical band gap values were determined from diffuse reflectance measurements, as described previously [27,28]. An example of the estimation of the optical band gap energy from UV-Vis DRS measurements is presented in Figure S3 in Supplementary Materials. The obtained data for all studied materials are collected in Figure 5. It can be seen that two optical band gaps can be determined for all samples. The first band gap energy of ~3.2 eV, observed for the material obtained after 5 s deposition, is related to the electronic transitions in the crystal structure of TiO_2_ nanotubes (anatase) [30]. For the extended deposition process, band gap narrowing is observed, probably due to a heterogeneous morphology of Fe_2_O_3_–TiO_2_ and the formation of a heterojunction, which was observed in our previous work [27,28]. On the other hand, a second energy band gap of ~2.2 eV located in the visible region of the solar spectrum was calculated for the studied samples and is related to the presence of hematite [31].

**Figure 6:**
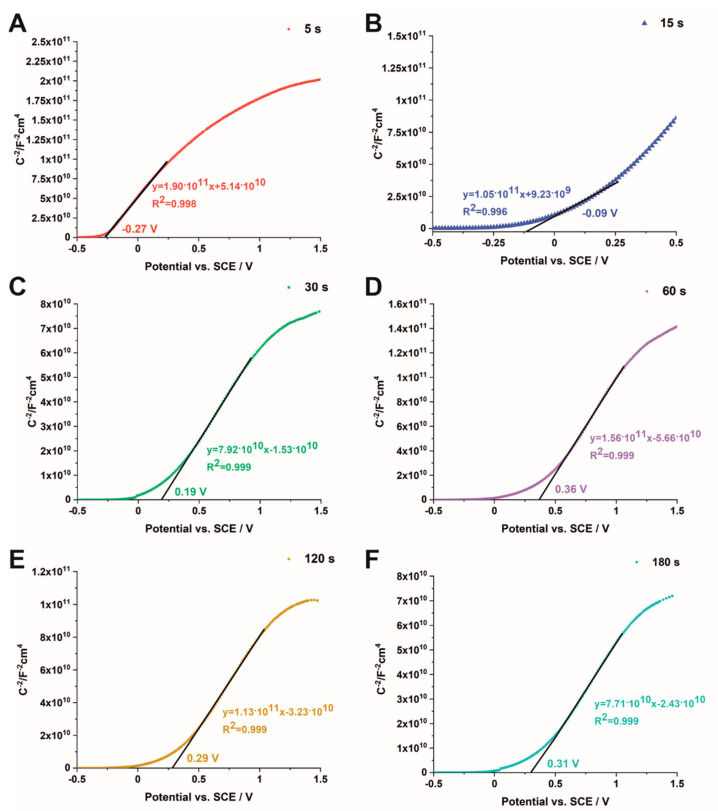
Mott–Schottky plots measured at 1000 Hz for the Fe_2_O_3_–TiO_2_ samples obtained by electrodeposition for 5 (**A**), 15 s (**B**), 30 s (**C**), 60 s (**D**), 120 s (**E**), and 180 s (**F**).

The semiconducting properties of Fe_2_O_3_–TiO_2_ materials were studied using Mott–Schottky analysis, according to the following equation (1) [22]:

(1) $$C_{SC}^{-2}=\left(\frac{2}{\varepsilon \varepsilon_{0}qN_{d}}\right)\left(E-E_{fb}-\frac{kT}{q}\right)$$

where: *C*_sc_ is the capacitance of the space-charge region (F cm^−2^), *N_d_* is the donor density (cm^−3^), *ɛ* is the dielectric constant of TiO_2_ (100), *ɛ*_0_ is the permittivity of free space (8.85∙10^−14^ F cm^−1^), *q* is the electron charge (1.602∙10^−19^ C), *E* is the applied potential (V), *E_fb_* is the flat band potential (V), *T* is the absolute temperature (K), and *k* is the Boltzmann constant (1.38∙10^−23^ J K^−1^) [32]. The Mott–Schottky analysis was performed at frequencies of 200, 500, and 1000 Hz. The flat band potential was obtained from the intersection of the *C_sc_*^−2^ vs. applied potential curve. The n-type behavior of the studied materials was confirmed by the positive slopes of the above-mentioned curves [33]. The Mott–Schottky plots measured at the frequency of 1000 Hz are presented in Figure 6 for samples obtained by electrodeposition for 5 s (A), 15 s (B), 30 s (C), 60 s (D), 120 s (E), and 180 s (F). As can be seen, with increasing the deposition time from 5 s to 180 s, the flat band potential shifts toward more positive potentials from −0.27 to 0.31 V vs. SCE, respectively. For the hybrid materials received by efigurelectrodeposition for 60 s, 120 s, and 180 s, the slight differences in *E_fb_* values are attributed to changes in the surface chemistry of TiO_2_ and concomitant modification of the surface states mediating the charge transfer across the material [34,35]. The above-mentioned results are consistent with the data in the literature, which typically report the flat band potential of hematite ranging between 0.16 V and 0.56 vs. SCE [36]. What is more, a good linear fit of the Mott–Schottky plot indicates a partially depleted space-charge layer at the semiconductor surface [37,38]. As an example, Mott–Schottky plots for three different frequencies (200, 500, and 1000 Hz) are shown in Figure S4 in Supplementary Materials for the sample electrodeposited for 5 s. As can be seen, the flat band potential of the hybrid material shifts from −0.27 to −0.02 V vs. SCE as the applied frequency changes from 1000 to 200 Hz, which can be attributed to the high crystallinity and porosity of the electrode as well as the presence of the metal component [22,27].

**Figure 7:**
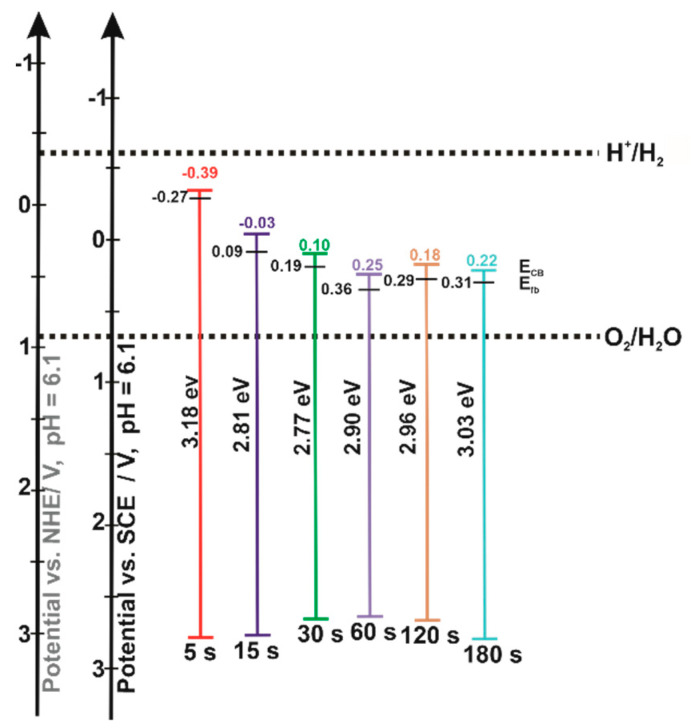
Energy positions of the conduction band (CB) and valence band (VB) of the studied Fe_2_O_3_–TiO_2_ samples.

The average donor densities of the studied samples were calculated based on Mott–Schottky measurements, as described earlier [27,28] Equation (S1) in Supplementary Materials for all tested frequencies (Figure S5, Supplementary Materials). As can be noticed, the donor density increases with increasing deposition time (except for the samples electrodeposited for 5 s and 30 s), which can be related to increasing the film thickness in a manner that depends on the substrate and its morphology, as suggested by Sellers and Seebauer [39]. The increased donor concentration reduces the width of the space-charge layer and, consequently, the electric field across the space-charge layer is larger. As a result, charge carriers within this region are efficiently separated and their recombination is inhibited [38]. It is worth noting that the dopant concentration should not be too high because it would provide more defect-scattering/recombination properties, which can equalize the increased separation efficiency [40]. Based on the flat band potentials and donor densities determined from the Mott–Schottky analyses, the conduction band edge was calculated for the studied samples from the following Equation (2) [41]:

(2) $$E_{CB}=E_{fb}+kT\cdot ln\frac{N_{sc}}{N_{CB}}$$

where: *E_CB_* is the conduction band edge (V), *E_fb_* is the flat band potential (V), *k* is the Boltzmann constant (8.62∙10^−5^ eV K^−1^), *T* is the semiconductor temperature (298 K), *N_sc_* is the donor density (cm^−3^), and *N_CB_* is the effective density of states in the conduction band (7.8∙10^20^ cm^−3^ [42]). The conduction band edges calculated for the analyses performed at 1000 Hz were −0.39, −0.03, 0.10, 0.25, 0.18, and 0.22 V vs. SCE for the samples electrodeposited for 5, 15, 30, 60, 120, and 180 s, respectively. The observed *E_CB_* shift toward more positive values with increasing electrodeposition time is consistent with the data in the literature and is strictly related to the formation of hematite layers [14]. Such a conduction band position of hematite causes a transfer of electrons from TiO_2_ to Fe_2_O_3_, leaving holes in the titanium oxide which may undergo oxidation reactions or act as recombination centers [14]. These results are opposite to those obtained by the impregnation method, where increasing the concentration of ferric chloride in the solution (5, 10, 25, 50, 100 mM) resulted in a slight shift in the conduction band edge toward more negative potentials [27], due to the much weaker effect of the hematite presence. To gain a deeper insight into the electronic properties of the studied hybrid Fe_2_O_3_–TiO_2_ materials, energy diagrams were constructed using band gap values determined from UV-Vis reflectance measurements and flat band potentials determined by Mott–Schottky analyses (Figure 7).

### 3.3. Photoelectrochemical Measurements

**Figure 8:**
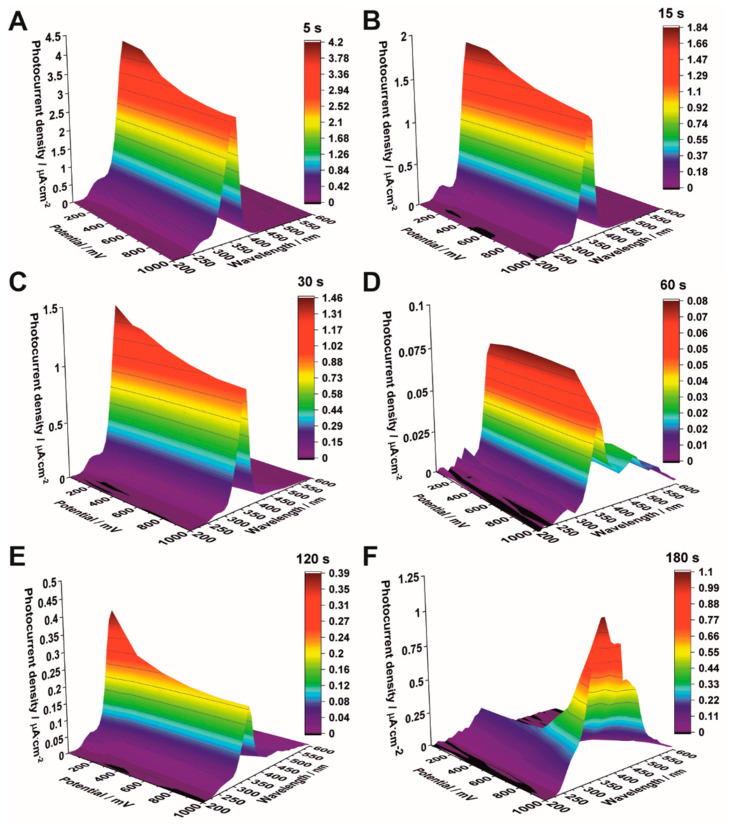
Photocurrent density vs. incident light wavelength and applied potential in 0.1 M KNO_3_ for Fe_2_O_3_–TiO_2_ samples obtained by electrodeposition for 5 s (**A**), 15 s (**B**), 30 s (**C**), 60 s (**D**), 120 s (**E**), and 180 s (**F**).

To evaluate the activity of the studied materials as potential photoelectrodes for water-splitting applications, photoelectrochemical properties were investigated under monochromatic conditions and solar radiation from a solar simulator with the Air-Mass 1.5G filter. The generated photocurrent under monochromatic radiation was recorded as a function of wavelength (300–600 nm) and potential (0–1 V vs. SCE) in 0.1 M KNO_3_ for all the studied samples (Figure 8). As can be seen, the highest photocurrent density was observed for the sample which was subjected to electrodeposition for 5 s (Figure 8A). The maximum photocurrent density was observed near 350 nm for all samples. However, the intensity of generated photocurrent gradually decreased in the UV range and simultaneously increased in the visible light range as the electrodeposition time was extended (Figure 8B–F). This behavior is closely related to the formation of hematite particles on the surface of anodic TiO_2_, which efficiently absorb visible light due to their narrow band gap (2.2 eV) [31].

**Figure 9:**
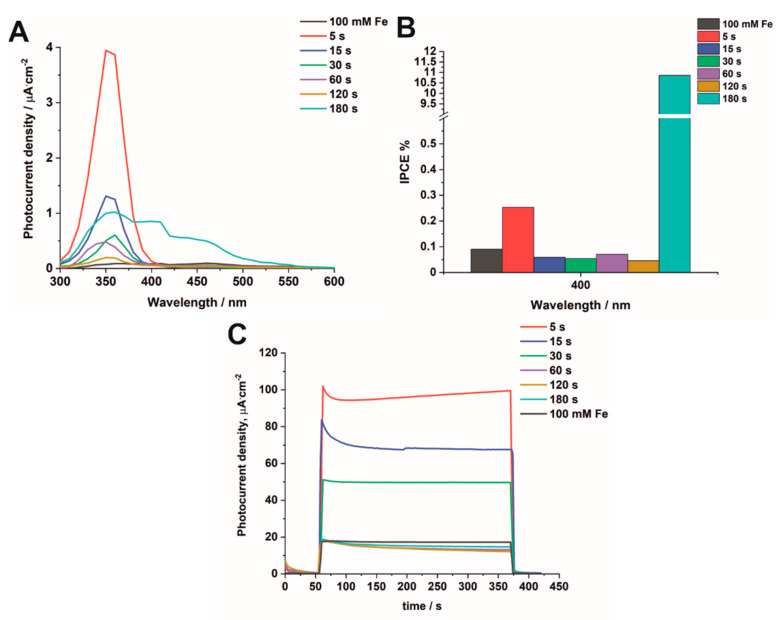
Photocurrent density vs. wavelength curves at 1 V vs. SCE in 0.1 M KNO_3_ under monochromatic radiation for Fe_2_O_3_–TiO_2_ samples obtained by electrodeposition for 5–180 s and impregnated in 100 mM FeCl_3_ (**A**). IPCE values obtained under monochromatic radiation for all modified samples at 400 nm (**B**). Photocurrent density vs. time curves at 1 V vs. SCE in 0.1 M KNO_3_ under solar radiation with the Air-Mass 1.5G filter for all modified samples (**C**).

Photocurrent density vs. illumination wavelength curves were recorded in 0.1 M KNO_3_ at 1 V vs. SCE under monochromatic radiation conditions (Figure 9A), and the obtained results are collected in Table S1 in Supplementary Materials. In close view, the sample electrodeposited for 5 s responds the most effectively to UV light irradiation due to the relatively small quantity of hematite particles deposited on the TiO_2_ surface. As the deposition time increases, more iron particles and, consequently, hematite are deposited on the TiO_2_ surface, which reduces the intensity of the photocurrent density in the UV range and increases it in the visible light region. The observed decrease in PEC response of the studied materials, with respect to increasing the iron content, is related to the low charge separation efficiency, which in turn is connected with a small hole diffusion length and short excited-state lifetime [43,44,45]. However, the sample with the highest amount of iron, and thus mostly covered with hematite particles, exhibited the highest photoresponse at the wavelength range of 450–500 nm, which is confirmed by the shape of the curve presented in Figure 9A. This PEC response in the visible light region is closely related to the hematite layer, having a narrow band gap (2.2 eV) and completely covering the surface of the anodic TiO_2_ [31].

From photocurrent density vs. time curves recorded for the Fe_2_O_3_–TiO_2_ samples electrodeposited for a longer period of time and impregnated (Figure S6, Supplementary Materials), it is clearly seen that the kinetics of photocurrent decay changes at higher Fe contents. Comparing the impregnated sample with that electrodeposited for 180 s, it is evident that the steady-state current conditions are gained within the time scale of ‘light on’ cycles for the impregnated sample, while the recombination of photogenerated charge carriers is observed during ‘light on’ cycles for the samples with a higher Fe content [45]. Additionally, for the sample electrodeposited for 180 s, characteristic cathodic current spikes appear when the light is turned off [46].

The incident photon-to-current efficiency (IPCE) values obtained at 1 V vs. SCE for 400 nm, 450 nm, and 500 nm were calculated as shown in Equation (S2) (Supplementary Materials) and presented in Figure 9B and Figure S7A,B and Table S1 in Supplementary Materials, respectively. It can be seen that the highest IPCE value at 400 nm, 450 nm, and 500 nm is observed for the sample electrodeposited for 180 s, which is associated with the formation of hematite absorbing visible light. The impregnated sample shows a similar IPCE value to the sample electrodeposited for 60 s due to a similar hematite content. For all studied samples, band gap values were also determined from PEC measurements as described previously [27,28]. An example of determining the energy band gap is given in Figure S8 in Supplementary Materials. The estimated band gaps are presented in Table S1 in Supplementary Materials. As expected, the lowest band gap was obtained for the sample electrodeposited for 180 s and for that impregnated in a 100 mM FeCl_3_ solution.

The kinetics of photocurrent decay were studied for the Fe_2_O_3_–TiO_2_ samples exposed to solar irradiation (Figure 9C), and the obtained results are presented in Table S1 (Supplementary Materials). As can be seen from Figure 9C, the anodic current peak related to the accumulation of charge near the surface of the semiconductor and exponential photocurrent decay to steady-state value are observed for the sample electrodeposited for 5 s. However, for longer deposition times (60–180 s), a steady-state photocurrent is reached quickly, almost immediately after the initiation of the ‘light on’ cycle. Among the samples electrodeposited for shorter durations (5–30 s), the sample electrodeposited for 5 s exhibited the best performance due to the highest photocurrent density generated under solar irradiation. It can be attributed to facilitated longitudinal electron transfer from the hematite to the Ti bulk through the TiO_2_ layer [10]. On the other hand, Fe_2_O_3_–TiO_2_ samples deposited for longer durations (60–180 s) contain hematite particles on the TiO_2_ surface and are characterized by a similar response, most probably caused by the presence of Fe^3+^ ions, which can act as recombination centers for photogenerated carriers [18].

### 3.4. Non-Enzymatic Glucose Sensing

The above-mentioned materials are typically used for photoelectrochemical and photocatalytic applications, such as water splitting [13], degradation of environmental pollutants [14,15,16], and solar cells [17,18]. However, an interesting recent application of semiconductor materials is their potential use as non-enzymatic glucose photosensors due to their self-cleaning properties [47]. What is more, the effective separation between the excitation source and the detection in the PEC shows ultra-sensitivity and a lower background signal, which might be crucial for biological and chemical analyses [48,49,50,51].

**Figure 10:**
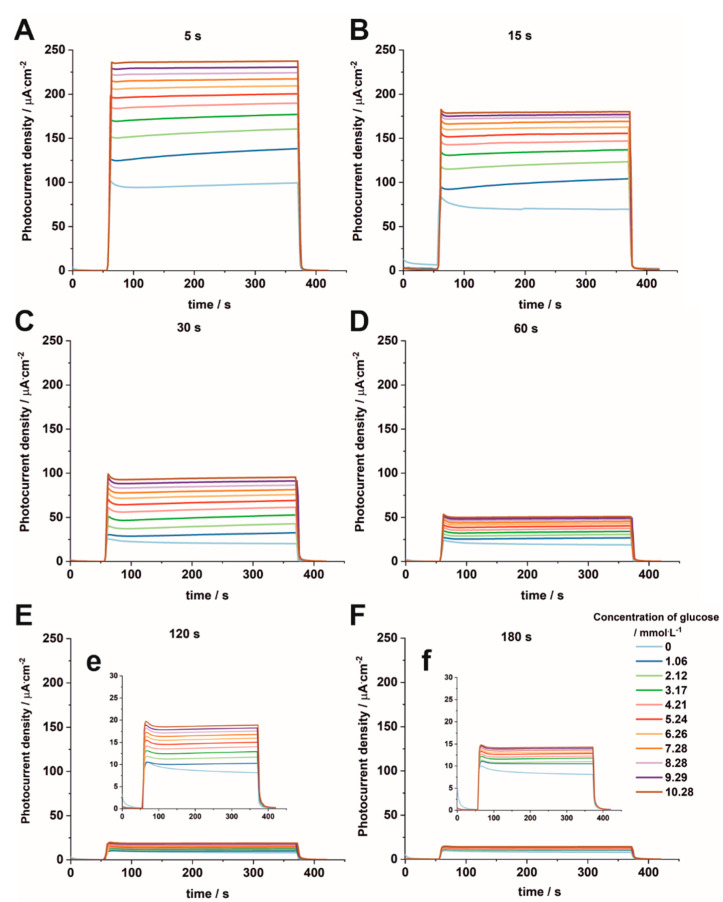
Photocurrent density vs. time curves measured at 1 V vs. SCE in 0.1 M KNO_3_ containing 1.06–10.28 mM of glucose under solar irradiation for samples deposited after 5 s (**A**), 15 s (**B**), 30 s (**C**), 60 s (**D**), 120 s (**E**), and 180 s (**F**), with insets (**e**) and (**f**).

To the best of our knowledge, there is not yet a clear answer in the literature as to whether hybrid Fe_2_O_3_–TiO_2_ materials are suitable as photoelectrodes for the electrochemical detection of glucose. Therefore, all studied samples were tested as photoelectrodes for glucose sensing. For this purpose, photocurrent density vs. time curves were recorded under solar irradiation at 1 V vs. SCE in a 0.1 M KNO_3_ solution in the presence of different concentrations of glucose for the studied materials (Figure 10). Moreover, the photoelectrochemical performance of the impregnated sample in glucose sensing was also presented for comparison (Figure S9, Supplementary Materials).

It can be seen from Figure 10 that the highest changes in the photocurrent with increasing concentrations of glucose are observed for Fe_2_O_3_–TiO_2_ electrodes obtained by electrodepositions carried out for 5 s (A) and 15 s (B). For samples electrodeposited for longer durations (Figure 10 C–F), the irradiated electrodes generate smaller photocurrent densities, but it can be still noticed that, in the solutions containing higher concentrations of glucose, the photocurrent density increases, such as for the impregnated sample. Additionally, the steady-state photocurrent is reached.

**Figure 11:**
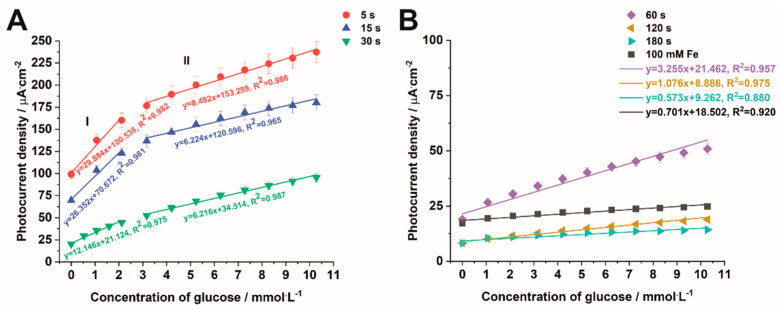
Calibration curves of photocurrent density against the concentration of glucose for Fe_2_O_3_–TiO_2_ electrodes obtained by electrodeposition carried out for 5–30 s (**A**) and for 60–180 s together with the sample impregnated in a 100 mM FeCl_3_ solution (**B**).

The calibration plots were constructed from the points at t = 350 s (with 5% error marked) from Figure 10. For samples deposited for 5 s to 30 s, two linear regions, namely I (at a low concentration range of 1.06–2.12 mM) and II (at a high concentration range of 3.17–10.28 mM), which demonstrated the best linearity, were fitted to the experimental data (Figure 11A). However, for samples deposited for a longer time and for the impregnated sample, one linear region can be found (Figure 11B). For the linear regions of the calibration plots, the sensitivity and the limit of detection (LOD = 3S_b_/m, where S_b_ is the standard deviation of the blank signal and m is the slope of the calibration curve), and the limit of quantification (LOQ = 10S_b_/m) were calculated. As the deposition time increases, the sensitivity of the method estimated for the lower concentration range (range I), as well for the higher concentration range (range II), decreases respectively, from 29.88 µA mM^−1^ cm^−2^ and 8.49 µA mM^−1^ cm^−2^ for the sample electrodeposited for 5 s to 12.15 µA mM^−1^ cm^−2^ and 6.22 µA mM^−1^ cm^−2^ for the sample electrodeposited for 30 s. For the samples electrodeposited for 60–180 s, the sensitivity decreases from 3.26 µA mM^−1^ cm^−2^ to 0.57 µA mM^−1^ cm^−2^. It can be seen that the highest sensitivities were found for the electrodes prepared for the samples electrodeposited for 5 s and 15 s, and they were even better than those obtained by Liu et al. [52] for Fe_2_O_3_ nanoparticles on fluorine-doped indium oxide (FTO), which are equal to 17.23 µA mM^−1^ cm^−2^. The calculated LOD and LOQ values are collected in Table S2 in Supplementary Materials. It is worth noticing that both LOD and LOQ values decrease with increasing electrodeposition time used for the formation of iron oxide particles on the TiO_2_ surface. Taking into account the above-mentioned characteristics of the tested electrodes, namely the sensitivity, LOD, and LOQ values, the best electrode for PEC detection of glucose turned out to be that prepared from the sample electrodeposited for 5 s. A comparison of the results obtained for the sample electrodeposited for 5 s with those found in the literature for different kinds of Fe_2_O_3_ materials [52,53,54,55,56] is presented in Table S3 in Supplementary Materials. The proposed sensor is distinguished by its sensitivity compared to those in other works.

## 4. Conclusions

In this research, it was shown that the iron content in the tested Fe_2_O_3_–TiO_2_ materials was in the range of ca. 0.5–30 at.%, depending on the electrodeposition duration. XRD and Raman spectroscopy measurements confirmed the formation of hematite based TiO_2_ materials. The Mot–-Schottky analyses revealed that flat band potential shifts toward more positive values, which suggests that water oxidation should be facilitated. Based on UV-Vis DRS measurements, two optical energy band gaps were estimated. One, which changes from 3.18 eV to 2.77 eV as deposition time increases to 30 s, and from 2.90 eV to 3.03 eV for longer durations, and an additional energy band gap of about 2.2 eV, appeared due to the presence of hematite. It was noticed that the samples exposed to monochromatic light responded differently in comparison to solar irradiation. The best PEC activity under irradiation with monochromatic UV light and solar light showed the sample deposited for 5 s, due to a low hematite content. However, the best photoresponse under visible light was observed for the sample deposited for 180 s, which is related to the presence of the hematite covering the surface. It was demonstrated that the studied Fe_2_O_3_–TiO_2_ materials are sensitive to the photoelectrochemical detection of glucose and can be used for the preparation of PEC sensors operating under solar radiation.

## Figures and Tables

**Table 1:** Procedures for the synthesis of Fe/Fe_2_O_3_–TiO_2_ materials by electrochemical deposition of iron/iron oxides in anodic TiO_2_ nanotubes (NTs). Dp—pore diameter (inner); Dc—interpore distance (outer); L—length; W—wall thickness; A—anodic; C—cathodic; P—pulsed; PC—potential cycling.

Morphology of TiO_2_ NTs [nm]	Pre-Treatment (Immersion)	Electrochemical Deposition Conditions				Post-Treatment	Obtained Material	Ref.
		Electrolytic Bath	Temperature [°C]	Time [s]	Technique/Conditions			
Dp = 80 L ≈ 550 W = 15–20	-	aqueous solution of 60 g FeSO_4_, 1.5 g C_6_H_8_O_6_, 0.5 g H_2_NSO_3_H, 15 g H_3_BO_3_	ambient	60–300	**P** cathodic current pulse: 8 ms of −70 mAcm^−2^; anodic potential pulse: 2 ms of 3 V	**Annealing** 500 °C for 6 h in an oxygen atmosphere	α-Fe_2_O_3_–TiO_2_ NTs	[11]
-	-	0.02 M FeCl_3_ in water:glycerol mixture (volume ratio: 1:1)	ambient	300–3600	**C** potential: −1 V, −3 V, −5 V	**Annealing** 450 °C for 2 h in air	α-Fe_2_O_3_–TiO_2_ NTs	[12]
Dp = 100 L = 400–500	5 mM NaF, 0.1 M NaCl, 1 M H_2_O_2_, 5 mM FeCl_3_ for 1 h, and 24 h	5 mM NaF, 0.1 M NaCl, 1 M H_2_O_2_, 5 mM FeCl_3_	-	-	**PC** potential: −0.52 V to 0.41 V vs. SCE, sweep rate: 0.1 V s^−1^, number of cycles: 50	**Annealing** 500 °C for 30 min in air	Fe_2_O_3_@TiO_2_ NTs	[13]
Dp = 80–100 W = 15 – 20 Dp ≈ 80 Dc ≈ 110 L ≈ 1200	0.05 M Fe(NO_3_)_3_ for 10 min	0.1 M Na_2_SO_4_	85	1200	**A** potential: 8 V	**Electrochemical oxidation** of Fe/TiO_2_-NT at 8 V for 2 min at room temperature in 1 M KOH	Fe_2_O_3_/TiO_2_ NTs	[14,15]
Dc = 270 L = 3300	-	0.2 M Fe(NO_3_)_3_	25	-	**PC** potential: 0 to −0.5 V vs. Ag/AgCl, scan rate: 0.01 V s^−1^, number of cycle: 2	-	Fe–TiO_2_ NTs	[16]
Dp ≈ 75 L = 1000	-	0.1 M FeSO_4_, 0.1 M H_3_BO_3_ (pH 3.5)	-	20–160	**C** potential: −1.3 V vs. SCE	**Annealing** 450 °C for 3 h in air	Fe_2_O_3_/TiO_2_-NTs	[17]
Dp = 54 ± 9 Dc = 93 ± 11 W = 18 ± 5	-	0.1 M FeCl_3_	-	600	**A** potential: 2 V, 2.5 V, 3 V, 4 V	**Annealing** 500 °C for 2 h	Fe–TiO_2_ NTs	[18]

## Data Availability

Not applicable.

## Supplementary Materials

The following Supplementary Materials can be downloaded at https://www.mdpi.com/article/10.3390/nano12091546/s1: Figure S1, Current density-time curves recorded during Fe deposition at -1.3 V vs. SCE for different durations (5–180 s) (A). The corresponding total charge densities obtained from current-time transients (B); Figure S2, Raman spectra of the Fe_2_O_3_–TiO_2_ sample electrodeposited for 60 s. The colors of different areas at the image correspond to the colors of the Raman spectra; Figure S3, Example of determination of the energy band gap from UV-Vis measurements together with a diffuse reflectance plot (a); Figure S4, Mott–Schottky plots measured at different frequencies (1000, 500, and 200 Hz) for the sample electrodeposited for 5 s; Figure S5, Average donor densities estimated for all studied materials, and for all tested frequencies; Figure S6, Photocurrent density vs. time curves recorded at 1 V vs. SCE for Fe_2_O_3_–TiO_2_ samples obtained by electrodeposition for 60 s and 180 s as well as by impregnation method; Figure S7, IPCE values obtained under monochromatic radiation for all modified samples at 450 nm (A) and 500 nm (B); Figure S8, Example of determining the energy band gap from photoelectrochemical measurements for the Fe_2_O_3_–TiO_2_ sample obtained by electrodeposition for 30 s; Figure S9, Photocurrent density vs. time curves measured at 1 V vs. SCE in 0.1 M KNO_3_ containing 1.06–10.28 mM of glucose under solar irradiation of the Fe_2_O_3_–TiO_2_ sample impregnated in 100 mM FeCl_3_ with inset; Table S1, The comparison of PEC properties of Fe_2_O_3_–TiO_2_ materials measured in 0.1 M KNO_3_ at 1 V vs. SCE; Table S2, Calculated LOD and LOQ values for all studied Fe_2_O_3_–TiO_2_ electrodes; Table S3, Glucose-sensing characteristics of the proposed sensor compared with data in the literature.
