# Supplementary material for: Photoelectrochemical Performance of Nanotubular Fe2O3–TiO2 Electrodes under Solar Radiation
Source: Nanomaterials (Basel). 2022 May 3;12(9):1546. doi: 10.3390/nano12091546 (PMC9131132; doi:10.3390/nano12091546)
Supplement: Supplementary file 1 [file nanomaterials-12-01546-s001.zip › nanomaterials-1683132-SI.pdf]

nanomaterials

MDPI

Article

Photoelectrochemical Performance of Nanotubular Fe_2O_3–TiO_2 Electrodes under Solar Radiation

Monika Sołtys-Mróz *, Karolina Syrek *, Łukasz Pięta, Kamilla Malek and Grzegorz D. Sulka

Faculty of Chemistry, Jagiellonian University, Gronostajowa 2, 30-387 Krakow, Poland; lukasz.pieta@doctoral.uj.edu.pl (Ł.P.); kamilla.malek@uj.edu.pl (K.M.); sulka@chemia.uj.edu.pl (G.D.S.)
* Correspondence: soltys@chemia.uj.edu.pl (M.S.-M.); syrek@chemia.uj.edu.pl (K.S.); Tel.: +48-12-686-25-73 (M.S.-M.)

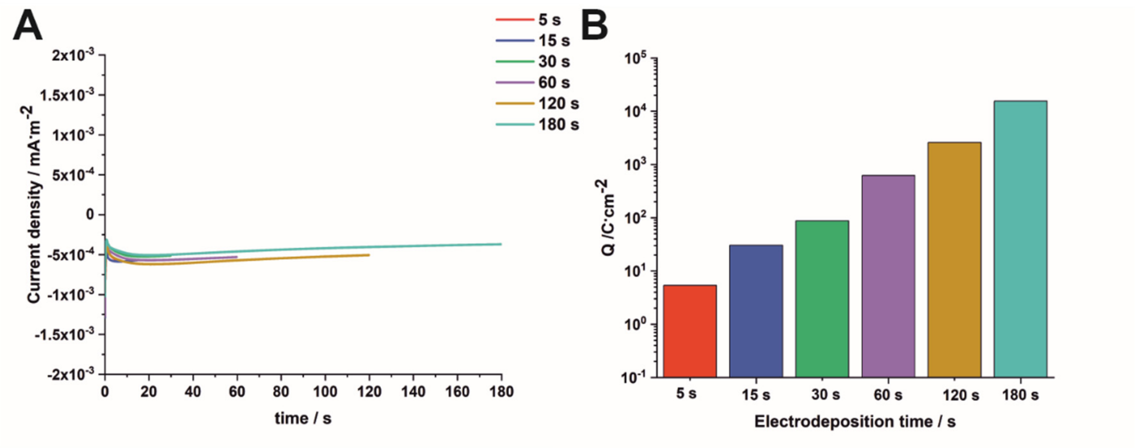

Figure S1. Current density-time curves recorded during Fe deposition at −1.3 V vs. SCE for different durations (5–180 s) (**A**). The corresponding total charge densities obtained from current-time transients (**B**).

Raman Spectroscopy

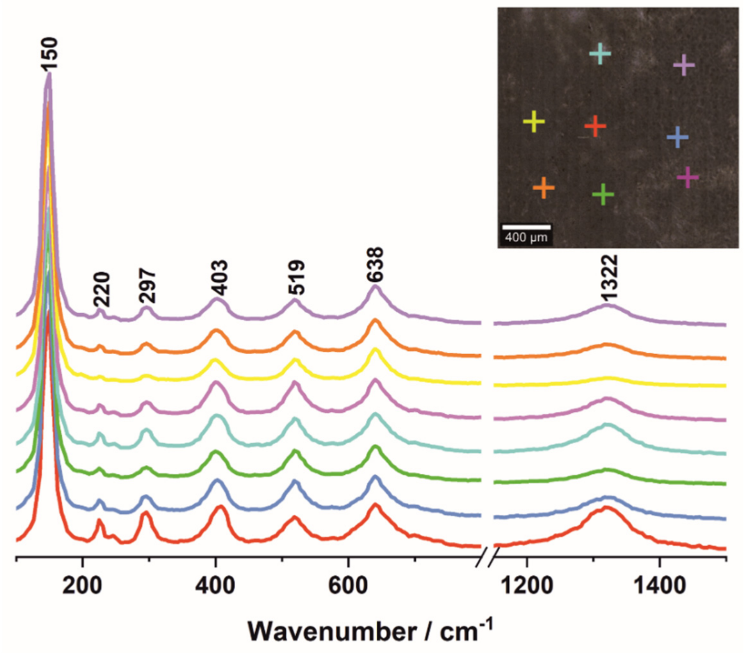

Figure S2. Raman spectra of the Fe_2O_3-TiO_2 sample electrodeposited for 60 s. The colors of different areas at the image correspond to the colors of the Raman spectra.

Semiconducting Properties

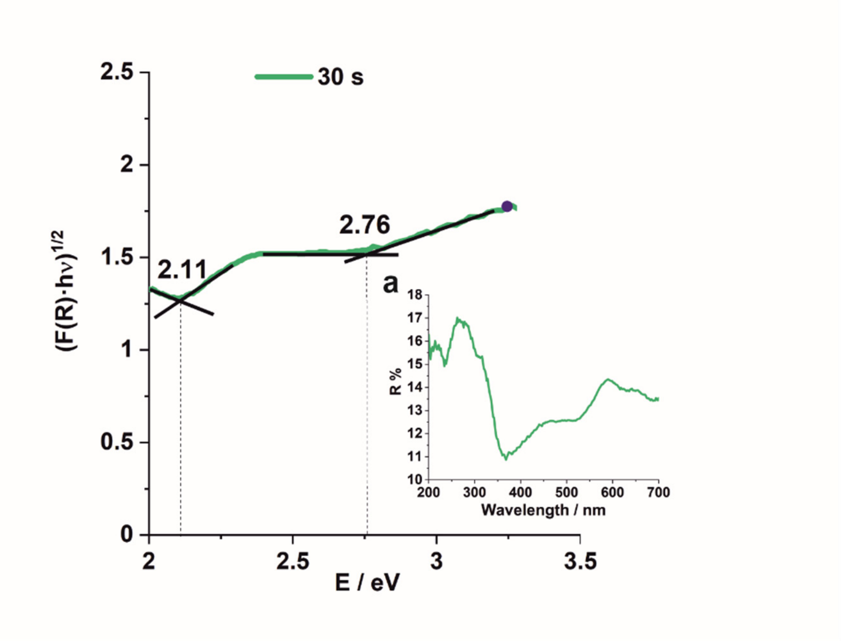

Figure S3. Example of determination of the band gap energy from UV-Vis measurements together with a diffuse reflectance plot (a).

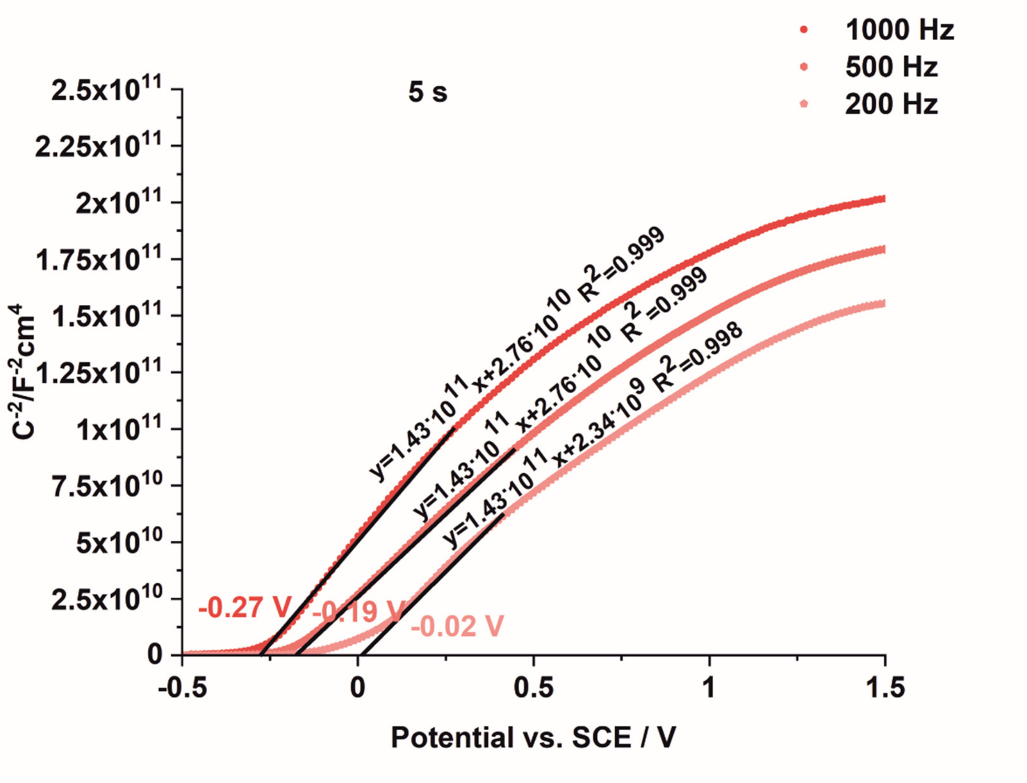

Figure S4. Mott-Schottky plots measured at different frequencies (1000, 500, and 200 Hz) for the sample electrodeposited for 5 s.

The donor density (N_d) for nanotubular TiO_2 and hybrid Fe_2O_3-TiO_2 materials was determined as follows (S1):

$$N_d = \frac{2}{\varepsilon_0 \varepsilon_S A^2 e \; slope} \quad \text{(S1)}$$

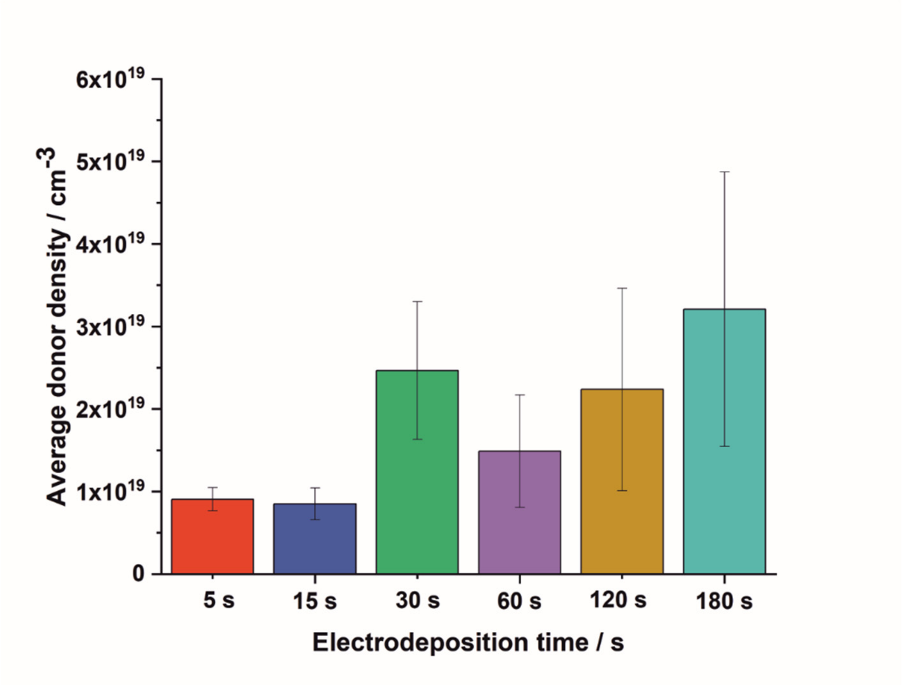

Figure S5. Average donor densities estimated for all studied materials, and for all tested frequencies.

Photoelectrochemical Measurements

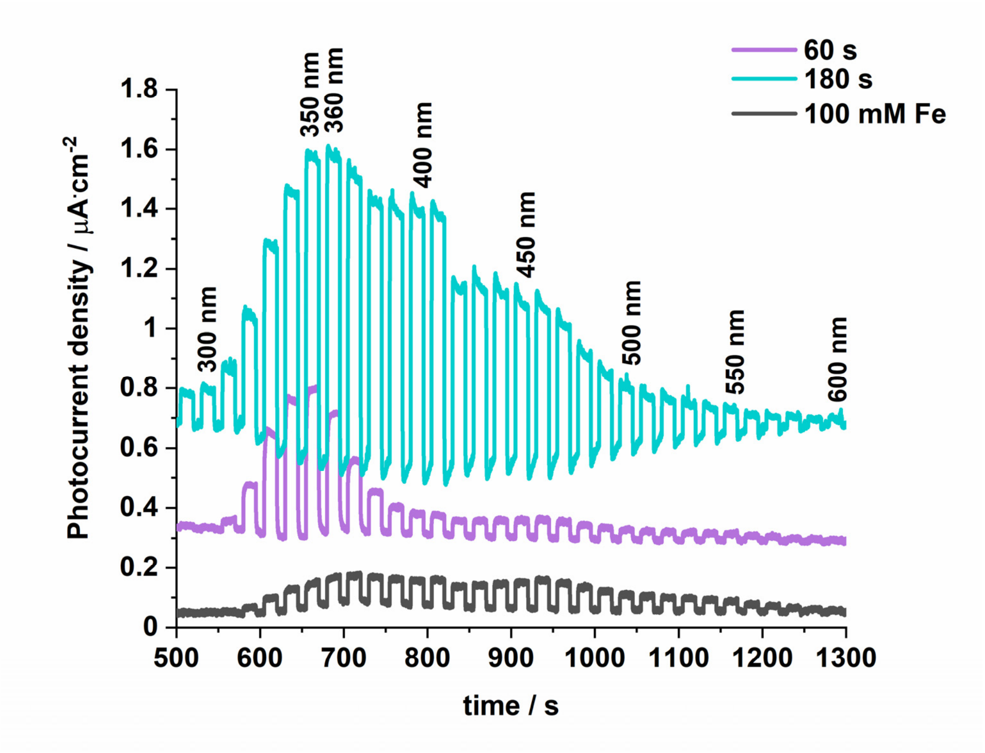

Figure S6. Photocurrent density vs. time curves recorded at 1 V vs. SCE for Fe_2O_3-TiO_2 samples obtained by electrodeposition for 60 s and 180 s as well as by impregnation method.

The Incident Photon to Current Efficiency (IPCE) was calculated based on the following formula (S2) [27]:

$$\mathrm{IPCE} = 1240\,\frac{I_P(\lambda)}{P(\lambda)\lambda} \tag{S2}$$

where: 1240 is a constant (W nm A^{-1}), I_P is the photocurrent density (A m^{-2}) at the wavelength λ (nm), and P is the incident power density of light (W m^{-2}) at λ.

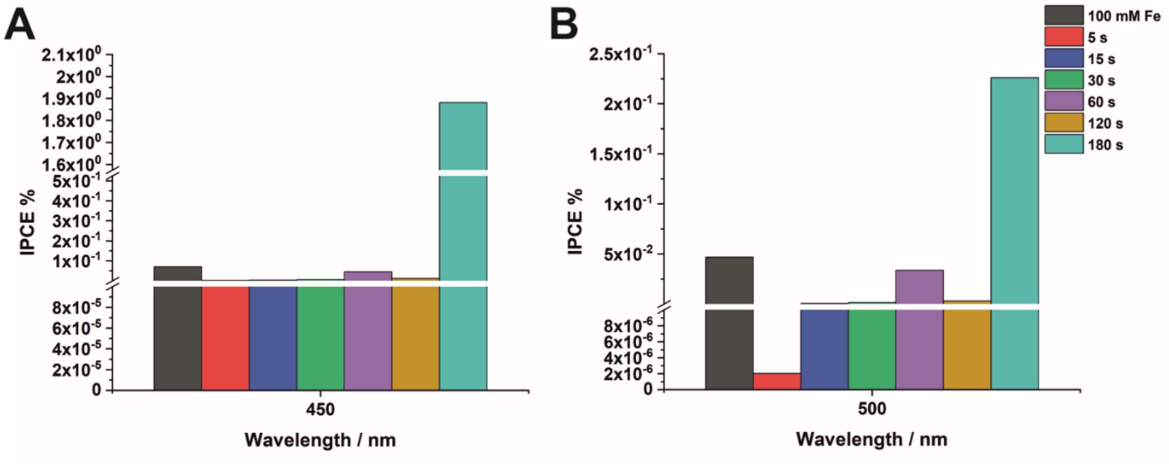

Figure S7. IPCE values obtained under monochromatic radiation for all modified samples at 450 nm (A) and 500 nm (B).

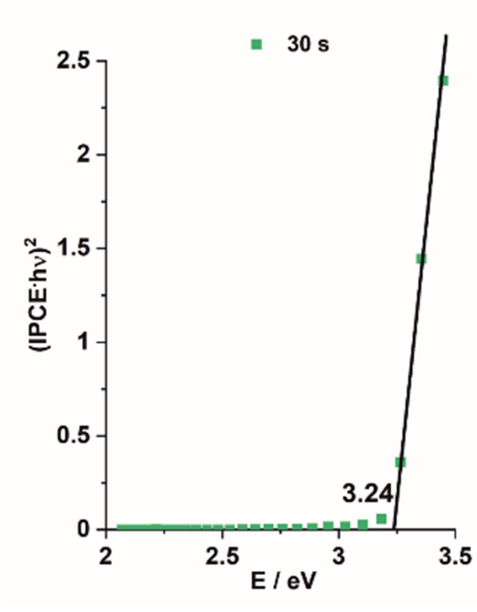

Figure S8. Example of determining the energy band gap from photoelectrochemical measurements for the Fe_2O_3-TiO_2 sample obtained by electrodeposition for 30 s.

Table S1. The comparison of PEC properties of Fe_2O_3-TiO_2 materials measured in 0.1 M KNO_3 at 1 V vs. SCE.

Samples (Electrodeposition Time or Impregnation Solution Concentration)	Photocurrent Density under Monochromatic Radiation [μA·cm^{-2}]	IPCE %			Photocurrent Density under Solar Radiation [μA·cm^{-2}]	Band Gap Energy [eV]
		400 nm	450 nm	500 nm		
**5 s**	3.9×10^{0} **/350 nm**	2.5×10^{-1}	1.2×10^{-3}	2.0×10^{-6}	9.9×10^{1}	3.22 ± 0.01
**15 s**	1.3×10^{0} **/350 nm**	5.9×10^{-2}	2.9×10^{-3}	4.1×10^{-4}	6.8×10^{1}	3.23 ± 0.01
**30 s**	6.0×10^{-1} **/360 nm**	5.4×10^{-2}	5.9×10^{-3}	1.3×10^{-3}	5.0×10^{1}	3.17 ± 0.01
**60 s**	4.8×10^{-1} **/350 nm**	7.1×10^{-2}	4.5×10^{-2}	3.4×10^{-2}	1.3×10^{1}	3.12 ± 0.01
**120 s**	2.0×10^{-1} **/350 nm**	4.5×10^{-2}	1.2×10^{-2}	3.1×10^{-3}	1.2×10^{1}	3.16 ± 0.01
**180 s**	1.0×10^{0} **/360 nm**	1.1×10^{1}	1.9×10^{0}	2.3×10^{-1}	1.5×10^{1}	2.80 ± 0.01
**100 mM**	8.8×10^{-2} **/360 nm**	9.0×10^{-2}	7.1×10^{-2}	4.7×10^{-2}	1.7×10^{1}	2.73 ± 0.01

Non-Enzymatic Glucose Sensing

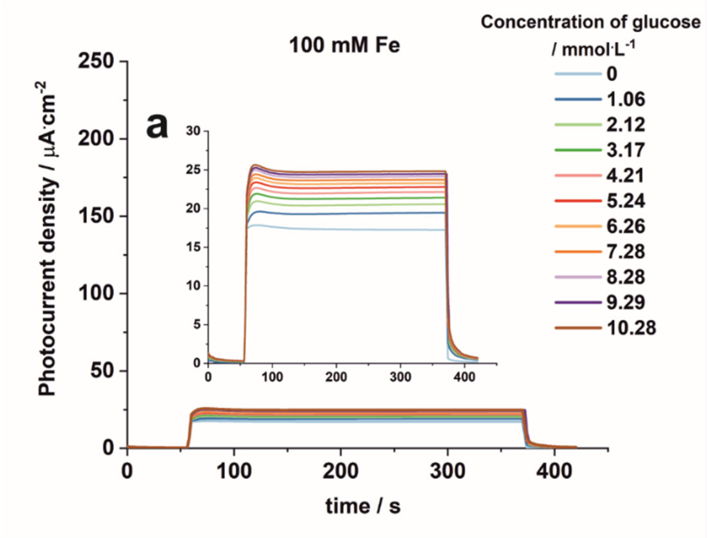

Figure S9. Photocurrent density vs. time curves measured at 1 V vs. SCE in 0.1 M KNO_3 containing 1.06–10.28 mM of glucose under solar irradiation of the Fe_2O_3-TiO_2 sample impregnated in 100 mM $FeCl_3$ with inset.

Table S2. Calculated LOD, and LOQ values for all studied Fe_2O_3-TiO_2 electrodes.

Samples (Electrodeposition Time or Impregnation Solution Concentration)	LOD [mM L⁻¹]		LOQ [mM L⁻¹]	
	I Concentration Range	II Concentration Range	II Concentration Range	II Concentration Range
**5 s**	14.806	8.686	49.354	28.954
**15 s**	14.075	5.227	46.915	17.424
**30 s**	24.175	8.860	80.583	29.533
**60 s**	7.444		24.813	
**120 s**	9.669		32.230	
**180 s**	4.058		13.526	
**100 mM Fe**	5.053		16.843	

Table S3. Glucose-sensing characteristics of the proposed sensor compared with data in the literature.

Material	Sensitivity [μAmM^{-1}·cm^{-2}]	Linear Range [mM]	Detection Limit [μM]	Response Time [s]	Reference
Fe_2O_3 nanowires	726.9	0.015–8	6	< 6	[53]
Fe_2O_3 cubes/ITO	32.3	0.2–2	0.015	-	[54]
Fe_2O_3-graphene	-	0.0005–0.01	0.5	-	[55]
Fe_2O_3 films/FTO	17.23	0.05–6	0.05	< 5	[52]
Fe_2O_3NR/FTO	100.46	0.2–2.0	5.5	-	[56]
Fe_2O_3-TiO_2 nanotubes	29.88	1.06–2.12<br>3.17–10.28	14810<br>8690	-	This work
